# High expression of KPNA2 defines poor prognosis in patients with upper tract urothelial carcinoma treated with radical nephroureterectomy

**DOI:** 10.1186/s12885-015-1369-8

**Published:** 2015-05-09

**Authors:** Bentao Shi, Boxing Su, Dong Fang, Yuan Tang, Gengyan Xiong, Zhongqiang Guo, Qun He, Xinyu Yang, Wei Zhao, Yinglu Guo, Xuesong Li, Liqun Zhou

**Affiliations:** 1 Department of Urology, Peking University Shenzhen Hospital, Shenzhen, Guangdong 518036 China; 2 National Urological Cancer Center, Beijing& Institute of Urology, Peking University, Beijing, 100034 China; 3 Department of Urology, Peking University First Hospital, Beijing, 100034 China; 4 Department of Urological Pathology, Peking University First Hospital & Institute of Urology, Peking University, Beijing, 100034 China; 5 Department of Cell Biology, Peking University School of Oncology, Beijing Cancer Hospital and Institute, Beijing, 100142 China

**Keywords:** KPNA2, Upper tract urothelial carcinoma, Prognosis, Bladder recurrence

## Abstract

**Background:**

To analyze the expression of karyopherin alpha 2 (KPNA2) in upper tract urothelial carcinoma (UTUC) and to investigate whether the KPNA2 expression provides additional prognostic information following radical nephroureterectomy (RNU).

**Methods:**

A tissue microarray (TMA) containing samples from 176 patients with UTUC who underwent RNU at our institute was analyzed for KPNA2 expression using immunohistochemistry. KPNA2 expression in normal urothelial cell line and urothelial carcinoma cell lines was evaluated by western blot analysis. Using RNA interference *in vitro*, the effects of KPNA2 inhibition on cellular viability, migration and apoptosis were determined.

**Results:**

KPNA2 expression was significantly upregulated in the UTUC samples compared with the adjacent normal urothelial tissues. High KPNA2 immunoreactivity was identified as a predictor of bladder recurrence (hazard ratio [HR]: 2.017, 95% CI 1.13-3.61, *p* = 0.018), poor disease-free survival (DFS, HR: 2.754, 95% CI 1.68-4.51, *p* = 0.001) and poor overall survival (OS, HR: 4.480, 95% CI 1.84-10.89, *p* = 0.001) for patients with UTUC after RNU. Furthermore, high KPNA2 immunoreactivity was independent of the conventional predictive factors in a multivariate analysis. Additional *in vitro* experiments revealed that KPNA2 expression was higher in urothelial carcinoma cell lines than in normal urothelial cell line. KPNA2 inhibition with a specific siRNA decreased cell viability and migration and increased apoptosis in urothelial carcinoma cell lines.

**Conclusions:**

KPNA2 is a novel independent prognostic marker for bladder recurrence, DFS and OS of UTUC patients who have undergone RNU. Moreover, these data suggest that KPNA2 may be a promising therapeutic target for UTUC.

## Background

Urothelial carcinomas are the fourth most common tumors after prostate (or breast), lung and colorectal cancer. This carcinoma is derived from the urothelium of the upper urinary tract (renal pelvis and ureter) or lower urinary tract (urinary bladder). In contrast with bladder urothelial carcinomas, upper urinary tract urothelial carcinomas (UTUCs) are relatively rare and account for only 5 to 10% of urothelial carcinomas [1] A previous study found that the ratio of urothelial carcinoma incidence in the renal pelvis, ureter, and urinary bladder is approximately 3:1:51 [2].Radical nephroureterectomy (RNU) with excision of the bladder cuff is the standard procedure for UTUC [3]. However, tumor recurrence remains common; specifically, the disease recurs in the bladder in 22–47% of UTUC patients [4]. The clinical characteristics and prognosis are different for UTUC and bladder cancer. The upper urinary tract has specific relevant anatomical characteristics, including a thin muscle layer, proximity to the kidney and rich lymphatic drainage. Tumor invasion may significantly influence distant metastasis and progression in UTUC patients. Approximately 60% of UTUCs are invasive at diagnosis, in contrast to only 15-25% of bladder cancers [5,6].

To date, the prognostic factors for recurrence and survival of patients after RNU remain unclear, and the published studies on prognostic factors for UTUC are limited and conflicting [7-9]. The clinicopathologic parameters of UTUC, such as tumor stage, histologic grade, and lymphovascular invasion (LVI), have been reported as independent predictors of clinical outcome following radical surgery [10].Some molecular markers, such as E-cadherin, hypoxia-inducible factor (HIF)-1α, snail, and Ki67 are also independently associated with tumor recurrence and poor survival [11]. However, none of the currently available markers have fulfilled the clinical and statistical criteria necessary to support their introduction into daily clinical decision making. Thus, identifying specific novel genes that can be effectively used as therapeutic targets and/or prognostic biomarkers is critical for the treatment of UTUC. However, only a relatively small number of studies have been completed to date.

KPNA2 belongs to the karyopherin (importin) family, which plays a fundamental role in nucleocytoplasmic transport [12]. Together with importin-β, KPNA2 delivers numerous cargo proteins to the nucleus, as guided by a nuclear localization signal that may also be important for oncogenesis [13]. KPNA2 has been identified and validated as a potential biomarker for many cancers, such as non-small cell lung cancer [14], breast cancer [15], ovarian cancer [16], and prostate cancer [17]. High expression of KPNA2 was investigated as an independent predictor of poor prognosis in patients with non–muscle-invasive bladder cancer and in patients with invasive bladder cancer undergoing radical cystectomy [18].However, currently it is lack of relevant research about KPNA2 expression in UTCC .

In the present study, we analyzed the KPNA2 expression in UTUC tissues and the prognostic relevance of KPNA2 expression in patients with UTUC who had undergone RNU. Additionally, the role of KPNA2 in the proliferation, migration and apoptosis of urothelial carcinoma cell lines was analyzed *in vitro*.

## Methods

### Patient selection

This study was approved by the Review Board of the Peking University First Hospital (Beijing, P.R. China). Informed written consent was obtained from all patients. We retrospectively analyzed the clinical and pathologic data from 607 patients who underwent RNU for UTUC from January 2002 to December 2010 at our institute.176 patients with incomplete follow-up data, Among the remaining 431 patients with complete follow-up data, 255 were excluded from the study: 81 had concomitant/previous bladder tumors, 45 had bilateral synchronous UTUC, 34 underwent other surgeries instead of radical nephroureterectomy, 39 had a follow-up period of less than 12 months, 48 patients with a tumor less than 1.0 cm (diameter) (not suitable for formation of a tissue microarray), 8 had a positive surgical margin. At last the remaining 176 patients were included in this study, 81 cases with open surgery, 95 cases with laparoscopic surgery, All UTUC patients undergoing radical nephroureterectomy, not receiving routine lymphadenectomy. Only for those high risk patients, Such as preoperative imaging examination suspected lymph node metastasis, high stage and mulifocality, the routine lymphadenectomy was performed. None of the included patients received preoperative chemotherapy, although postoperative chemotherapy or radiotherapy was administered at the time of recurrence or metastasis. The clinical stage was assigned using the American Joint Committee on Cancer TNM Staging System for Renal Pelvis and Ureter Cancer (7th edition, 2010) [19].

In total, we observed 78 male and 98 female patients with a median age of 69 years (29–86) and a median follow-up of 36 months (12–108). The patients went to follow-up appointments on a routine schedule, including regular cystoscopy, urine cytology and clinical examinations. Cystoscopy was performed every three months for the first two years, then cystoscopy intervals were extended to 1 year thereafter. Chest X-ray, serum creatine, abdominal ultrasound, and computed tomography were examined at the same time. We defined bladder recurrence as finding a subsequent bladder tumor during cystoscopy and confirmed it by pathology. The time of first bladder recurrence was used as the endpoint for the study. The follow-up of patients without tumor recurrence was censored to the date of their last visit. In case of death not related to the bladder cancer, follow-up was censored to the date of death. Metastasis or retroperitoneal recurrence was documented.

### Immunohistochemistry (IHC) of tissue microarrays and scoring

The tissue microarrays were constructed as previously described [20].IHC was performed on formalin-fixed paraffin -embedded tissue microarrays (TMAs) sections that consisted of paired normal and UTUC specimens from 176 patients. IHC was completed using an SP reagent kit (Zhongshan Biotechnology Company, Beijing, China) according to the manufacturer’s instructions. An anti-KPNA2 antibody (1:800 Abcam plc, Cambridge, UK) was used as the primary antibody. For a negative control of the staining procedure, the primary antibody was omitted. Two surgical pathologists reviewed and scored the slides independently for KPNA2 expression without knowledge of the clinical data.

To analyze the KPNA2 staining, the percentage of cancer cells with nuclear immunoreactivity was quantified. Based on an analysis of different cut-off levels and previous studies [18,21], high expression of KPNA2 was defined as strong nuclear staining in at least 10% of the carcinoma cells.

### Cell lines

The normal urothelial cell line SV-HUC-1 and the urothelial transitional cell carcinoma cell lines 5637, RT4, T24, UM-UC-3 and J82 were obtained from the American Type Culture Collection. SV-HUC-1 was cultured in Ham’s F-12 medium. The 5637, RT4 and T24 cells were grown in RPMI 1640 medium. J82 and UM-UC-3 cells were maintained in DMEM. All culture media were supplemented with 10% fetal bovine serum (HyClone) and penicillin sodium (100 U/ml)/streptomycin sulfate (100 μg/ml) (Invitrogen). All cells were grown in a humidified atmosphere incubator with 5% CO_2_ at 37°C.

### RNA interference

Cells were transfected using the INTERFERINTM reagent (PolyplusTransfection, Strasbourg, France) according to the manufacturer’s instructions. A pool of two sequence-validated and knockdown-warranted KPNA2-siRNA was used (Homo-1111: 5′-GACUCAGGUUGUGAUUGAUTT-3′ and 5′-AUCAAUCACAACCUGAGUCTT-3′; homo-1400:5′-CCGUUGAUGAACCUCUUAATT-3′ and 5′-UUAAGAGGUUCAUCAACGGTT-3′) (GenePharma, Shanghai, China). Commercial FAM-tagged, negative control siRNAs (NC siRNA) (5′-UCCUCCGAACGUGUCACGUTT-3′,5′-ACGUGACACGUUCGGAGAATT-3′) (GenePharma) were used as an efficiency control and as a control for unspecific side effects. Cell lysates were prepared for western blotting 48 h after transfection to determine the efficiency of gene expression ablation.

### Western blot analysis

Total proteins from cell lines were extracted in lysis buffer (Thermo Fisher Scientific, Rockford, IL, USA) and quantified using a BCA protein assay (Thermo Scientific, Rockford, IL, USA). Each extracted protein sample was separated by 10% SDS-PAGE. After transferring the separated proteins to a PVDF membrane (Pall, Pensacola, FL), the membrane was incubated overnight at 4°C with antibodies against KPNA2 (Abcam Plc, Cambridge, UK, 1:1000), PARP (CST, Danvers, MA, 1:1000), PCNA (CST, Danvers, MA, 1:1000), or β-actin (Santa Cruz Biotechnology, 1:1000). After four washes with TBST, membranes were incubated with the appropriate HRP-conjugated secondary antibody at 37°C for 1 h. The protein bands were detected using Immobilon^TM^ Western Chemiluminescent HRP substrate (Millipore) and scanned using GeneSnap (Syngene, Cambridge, UK) acquisition software.

### Proliferation assays

The cell proliferation capacity of siRNA-transfected cultures was determined using Cell Counting Kit-8 solution (Dojindo, Gaithersburg, Kumamoto, Japan) according to the manufacturer’s protocol. Briefly, cells were seeded at a concentration of 5 × 10^3^ cells/100 μl/well in 96-well culture plates and treated with 10 μl/well of Cell Counting Kit-8H solution during the last 2 h of culturing. The optical density of the wells was measured at 450 nm using a Multiscan microplate spectrophotometer (Thermo LabSystems, Milford, MA).

### Migration assay

Cell migration was assessed using a Boyden chamber assay. A total of 5 × 10^4^ cells in 100 μl serum-free medium were seeded onto the upper chambers of a 24-well Boyden Chamber insert (Costar #3422) with uncoated 8-μm pores. Medium with 10% FBS was added to the lower chambers as a chemoattractant. After 24 h of incubation, cells remaining on the upper surface of the membrane were removed with a cotton swab, and cells that migrated through the membrane filter were fixed with 4% paraformaldehyde, stained with 0.1% crystal violet, and photographed under a microscope (Olympus BX40 with a DP70 digital camera). The migrating cells were manually counted per high-power field for each condition, and five fields were randomly selected per membrane.

### Apoptosis assay

Apoptosis was evaluated by using an Annexin-V/PI apoptosis detection kit (KeyGen Biotech, Nanjing, China) following the manufacturer’s instructions. Cells were cultured in 6-well plates at a concentration of 2-3 × 10^5^ cells/2 ml/well and transfected with siRNA. Cells were collected 48 h after transfection and then resuspended in 500 μl binding buffer, followed by the addition of 5 μl Annexin V-FITC and 5 μl PI dye. After an incubation of 10–15 min at room temperature in the dark, cells were analyzed using a BD FACStar flow cytometer (Becton Dickinson, San Jose, CA).

### Mitochondrial membrane potential (Δψm) assay

ΔΨm was estimated using a mitochondrial membrane sensor kit containing the cationic lipophilic fluorochrome JC-1 dye (KeyGEN Biotech, Nanjing, China) according to the manufacturer’s protocol. Briefly, 48 h after siRNA transfection, the cells were collected and resuspended in 500 μl JC-1 working solution for 20–30 min. Next, the cells were analyzed using a BD FACStar flow cytometer (Becton Dickinson, San Jose, CA).

### Caspase 3/7 activity assay

A total of 5 × 10^3^ cells were seeded in 96-well cell culture plates. After a 48 h siRNA treatment, apoptosis rates were measured based on the activation of effector caspases 3 and 7 using the Caspase-Glo^TM^3/7 Substrate kit (Promega, Mannheim, Germany) according to the manufacturer’s instructions. All samples were performed in triplicate.

### Statistical analyses

A two-sided Fisher’s exact test or Pearson’s 2-sided χ^2^ test was used to study significant differences between immunohistochemical and clinicopathologic data. To compare two independent samples, the nonparametric Mann–Whitney test was used. Survival curves for patients with low or high KPNA2 expression were plotted using the Kaplan-Meier method, with log-rank tests for statistical significance. Uni- and multi-variable Cox regression analyses were used to test the prognostic relevance of clinicopathologic/immunohistochemical data. Only predictive factors that were significant in the univariate analysis were used in the multivariate analysis (Cox’s proportional hazards model). SPSS version 17.0 (SPSS) was used to complete the statistical analysis. P < 0.05 was considered significant.

## Results

### Correlation between KPNA2 expression and clinicopathologic features of UTUC

**Figure 1:**
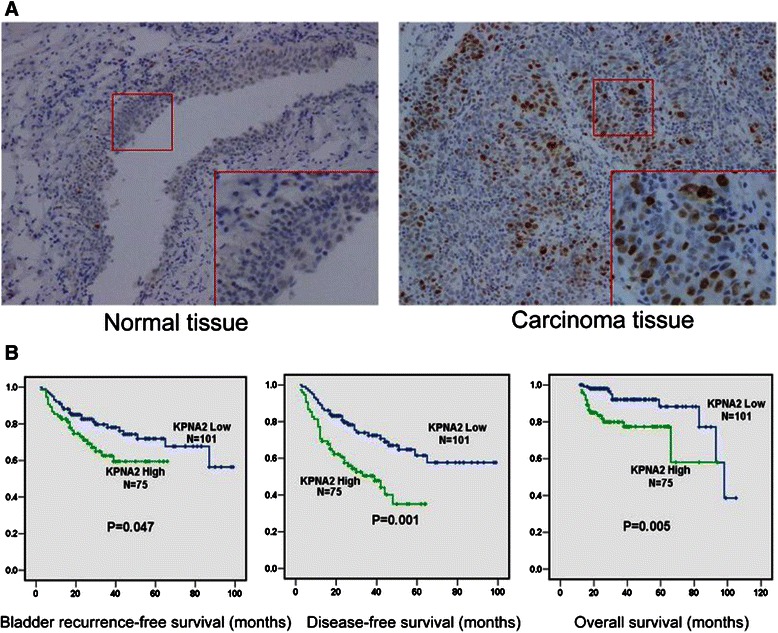
KPNA2 expression in normal and UTUC specimens and its correlation with survival. **(A)** Representative micrograph of IHC staining for KPNA2 in UTUC and adjacent normal tissues (Original magnification: ×100; ×200). **(B)** Kaplan–Meier curves of bladder recurrence-free survival, disease-free survival and overall survival of UTUC patients with high KPNA2 expression versus patients with low KPNA2 expression.

KPNA2 expression was investigated using immunohistochemical analysis of a TMAs containing 176 paired UTUC and adjacent normal specimens. Representative KPNA2 staining is shown in Figure 1A. KPNA2 expression is significantly higher in UTUC than in adjacent normal tissues. Table 1 lists the clinicopathologic features of patients and their correlation with KPNA2 expression in UTUC specimens. KPNA2 expression was significantly associated with sex (P = 0.038), T stage (*P <* 0.001) and G grade (*P <* 0.001).

**Table 1:** **Correlation between KPNA2 expression and clinicopathologic characteristics of UTUC patients after RNU**

		KPNA2 immunoreactivity		
Variable	Case No.	Low (N = 101)	High (N = 75)	*P* ^*^
Sex				0.038
Male	78	48	30	
Female	98	53	45	
Age (y)				0.95
≥70	84	48	36	
<70	92	53	39	
BMI (kg/m^2)^				0.26
>27	37	24	13	
27-22	81	46	35	
<22	58	31	27	
Tumor size (cm)				0.46
≥3.5	81	41	40	
<3.5	95	60	35	
T stage				<0.001
Ta-T1	61	48	13	
T2	70	36	34	
T3	42	17	25	
T4	3	0	3	
Pathologic lymph node stage				0.25
N0	168	98	70	
N+	8	3	5	
G grade				<0.001
G1	3	3	0	
G2	105	77	28	
G3	68	21	47	
eGFR (ml/min/1.73 m^2^)^#^				0.83
≥60	77	48	29	
60-15	90	49	41	
<15	9	4	5	
Hydronephrosis				0.24
Absent	70	46	24	
Present	106	65	41	
Location				0.30
	81	50	31	
Renal pelvis Proximal	6	5	1	
Middle	17	9	8	
Distal ureter	52	26	26	
Multiple	20	11	9	
Side				0.95
Left	82	49	33	
Right	94	52	42	
Tumor multiplicity				0.99
Single tumor focus	121	69	52	
Multiple tumor foci	55	32	23	
Tumor configuration				0.53
Papillary	140	82	58	
Nonpapillary	36	19	17	

*Mann–Whitney test for the comparison between two groups or Kruskal-Wallis test for more than two groups.

# Estimated glomerular filtration rate (eGFR) was calculated using the re-expressed Modification of Diet in Renal Disease (MDRD) formulas for the Chinese population.

### KPNA2 expression is a prognostic marker in UTUC patients after RNU

Using the log-rank test, Kaplan-Meier survival curves show an inverse correlation between KPNA2 expression and the patient survival rate (Figure 1B). The 5-year bladder recurrence-free survival rate for KPNA2-low expression patients (71.9%) was significantly higher than that for KPNA2-high expression patients (59.4%) (P = 0.047). The 5-year disease-free survival (DFS) rate for KPNA2-low expression patients (61.5%) was also significantly higher than the DFS rate for KPNA2-high expression patients (35.1%) (P = 0.001). The 5-year overall survival (OS) rate for KPNA2-low expression patients (88.2%) was also significantly higher than the OS rate for KPNA2-high expression patients (77.3%) (P = 0.005).

### High expression of KPNA2 is an independent prognostic factor for poor bladder recurrence-free survival of UTUC patients after RNU

Of the 176 patients, 50 patients (28.4%) developed subsequent bladder tumors at a median interval of 29 months (range: 2–99) after RNU. According to a univariate analysis, the predictive factors for bladder recurrence were age (P = 0.042), sex (P = 0.014), tumor location (P = 0.007), tumor side (P = 0.046), tumor multiplicity (P = 0.001), tumor stage (0.039) and KPNA2 expression (P = 0.001). The multivariate analysis revealed that sex (P = 0.017), tumor location (P = 0.015), tumor multiplicity (P = 0.002) and KPNA2 expression (P = 0.018) were significantly associated with bladder recurrence (Table 2) and were independent risk factors for bladder recurrence.However, tumor side was marginally significant (P = 0.057) in the multivariate analysis. Neither tumor stage nor tumor grade was an independent risk factor for bladder recurrence.

**Table 2:** **Predictive factors for bladder recurrence in UTUC patients after RNU**

Variable	Characteristics	Univariate		Multivariate	
		HR (95%CI)	P value	HR (95%CI)	P value
Age (y)	≥70 *vs <*70	0.969 (0.941-0.999)	0.042*	0.977 (0.953-1.003)	0.080
Sex	Male *vs* female	2.077 (1.157-3.729)	0.014*	1.980 (1.127-3.480)	0.017
BMI (kg/m^2^)	≥25 *vs <*25	0.791 (0.429-1.458)	0.452		
Tumor side	Left *vs* Right	1.853 (1.010-3.401)	0.046*	1.732 (0.984-3.049)	0.057
Tumor location	Ureter *vs* Pelvis	2.873 (1.342-6.151)	0.007	2.020 (1.147-3.560)	0.015
Tumor multiplicity	Multiple *vs* Single	2.770 (1.521-5.046)	0.001	2.431 (1.388-4.261)	0.002
Tumor size (cm)	≥3.5 *vs <*3.5	0.590 (0.311-1.119)	0.106		
Tumor configuration	Nonpapillary *vs* Papillary	0.592 (0.260-1.350)	0.212		
T stage	T2-T4 *vs* Ta-T1	0.951 (0.938-0.986)	0.039	0.970 (0.949-1.001)	0.075
G grade	High *vs* Low	0.696 (0.362-1.337)	0.277		
eGFR (ml/min/1.73 m^2^)	<60 *vs* ≥60	0.752 (0.397-1.422)	0.380		
Hydronephrosis	Present *vs* Absent	0.595 (0.270-1.309)	0.197		
Lymph node stage	N+ *vs* N0	0.226 (0.029-1.791)	0.159		
KPNA2 expression	High *vs* Low	3.052 (1.600-5.822)	0.001	2.017 (1.126-3.612)	0.018

HR, hazard radio; CI, confidence interval; BMI, body mass index; eGFR, estimated glomerular filtration rate.

### High expression of KPNA2 is an independent prognostic factor for the poor DFS and OS of UTUC patients after RNU

Of the 176 patients, 106 patients (64.2%) were disease-free and alive at a median follow-up of 39 months (range: 13–99). A total of 50 patients (28.4%) developed bladder recurrence, 12 patients (6.82%) developed non-bladder recurrence, and 20 patients (11.4%) developed metastasis. A total of 26 patients (14.8%) died after a median period of 20 months (range: 12–98). A univariate Cox regression analysis (Table 3) revealed that male sex (P = 0.004), tumors located in the ureter (P = 0.015), multiple tumor foci (P = 0.009),high stage(P = 0.012) and high KPNA2 expression (P < 0.001) were highly associated with a shorter DFS. In the multivariate Cox regression analysis, only high KPNA2 expression (P = 0.001), male gender (P = 0.002), multiple tumor foci (P = 0.013) and tumor stage(p = 0.022) remained significant. Male sex (P = 0.001), a tumor diameter larger than 3.5 cm (P = 0.005),,high stage(P = 0.001) and high KPNA2 expression (P = 0.001) were significantly associated with shorter OS both in the uni- and multi-variable Cox regression analyses (in Table 4).

**Table 3:** **Predictive factors for disease-free survival of UTUC patients after RNU**

Variable	Characteristics	Univariate		Multivariate	
		HR (95%CI)	P value	HR (95%CI)	P value
Age (y)	≥70 *vs <*70	0.996 (0.969-1.023)	0.761		
Sex	Male *vs* female	2.079 (1.268-3.409)	0.004	2.171 (1.340-3.516)	0.002
BMI (kg/m2)	≥25 *vs <*25	1.078 (0.648-1.791)	0.773		
Tumor side	Left *vs* Right	1.293 (0.782-2.137)	0.316		
Tumor location	Ureter *vs* Pelvis	2.243 (1.171-4.296)	0.015	1.526 (0.951-2.448)	0.080
Tumor multiplicity	Multiple *vs* Single	1.954 (1.180-3.234)	0.009	1.820 (1.132-2.925)	0.013
Tumor size (cm)	≥3.5 *v*s <3.5	1.293 (0.782-2.137)	0.316		
Tumor configuration	Nonpapillary *vs* Papillary	0.722 (0.369-1.414)	0.343		
T stage	T2-T4 *vs* Ta-T1	2.041 (1.173-4.195)	0.012	1.836 (1.132-3.125)	0.022
G grade	High *vs* Low	0.831 (0.471-1.467)	0.523		
eGFR (ml/min/1.73 m^2^)	<60 *vs* ≥60	0.822 (0.483-1.400)	0.471		
Hydronephrosis	Present *vs* Absent	0.552 (0.288-1.057)	0.073		
Lymph node stage	N+ *vs* N0	1.437 (0.458-4.512)	0.535		
KPNA2 expression	High *vs* Low	3.424 (1.972-5.944)	0.000	2.754 (1.683-4.506)	0.001

HR, hazard radio; CI, confidence interval; BMI, body mass index; eGFR, estimated glomerular filtration rate.

**Table 4:** **Predictive factors for overall survival of UTUC patients after RNU**

Variable	Characteristics	Univariate		Multivariate	
		HR (95%CI)	P value	HR (95%CI)	P value
Age (y)	≥70 vs <70	1.036 (0.981-1.095)	0.207		
Sex	Male vs female	4.195 (1.631-10.789)	0.003	4.155 (1.771-9.747)	0.001
BMI (kg/m2)	≥25 vs <25	0.933 (0.373-2.334)	0.882		
Tumor side	Left vs Right	0.482 (0.172-1.356)	0.167		
Tumor location	Ureter vs Pelvis	2.026 (0.524-7.836)	0.306		
Tumor multiplicity	Multiple vs Single	1.041 (0.398-2.724)	0.935		
Tumor size (cm)	≥3.5 vs <3.5	4.245 (1.526-11.803)	0.006	3.680 (1.495-9.056)	0.005
Tumor configuration	Nonpapillary vs Papillary	1.860 (0.676-5.116)	0.229		
T stage	T2-T4 vs Ta-T1	4.172 (1.601-10.535)	0.004	4.035 (1.678-9.521)	0.001
G grade	High vs Low	0.932 (0.360-2.411)	0.885		
eGFR (ml/min/1.73 m2)	<60 vs ≥60	1.247 (0.439-3.538)	0.678		
Hydronephrosis	Present vs Absent	1.081 (0.294-3.981)	0.907		
Lymph node stage	N+ vs N0	3.206 (0.715-14.378)	0.128		
KPNA2 expression	High vs Low	3.443 (1.271-9.329)	0.015	4.480 (1.844-10.887)	0.001

HR, hazard radio; CI, confidence interval; BMI, body mass index; eGFR, estimated glomerular filtration rate.

### KPNA2 knockdown reduces the viability and migration of urothelial carcinoma cells

**Figure 2:**
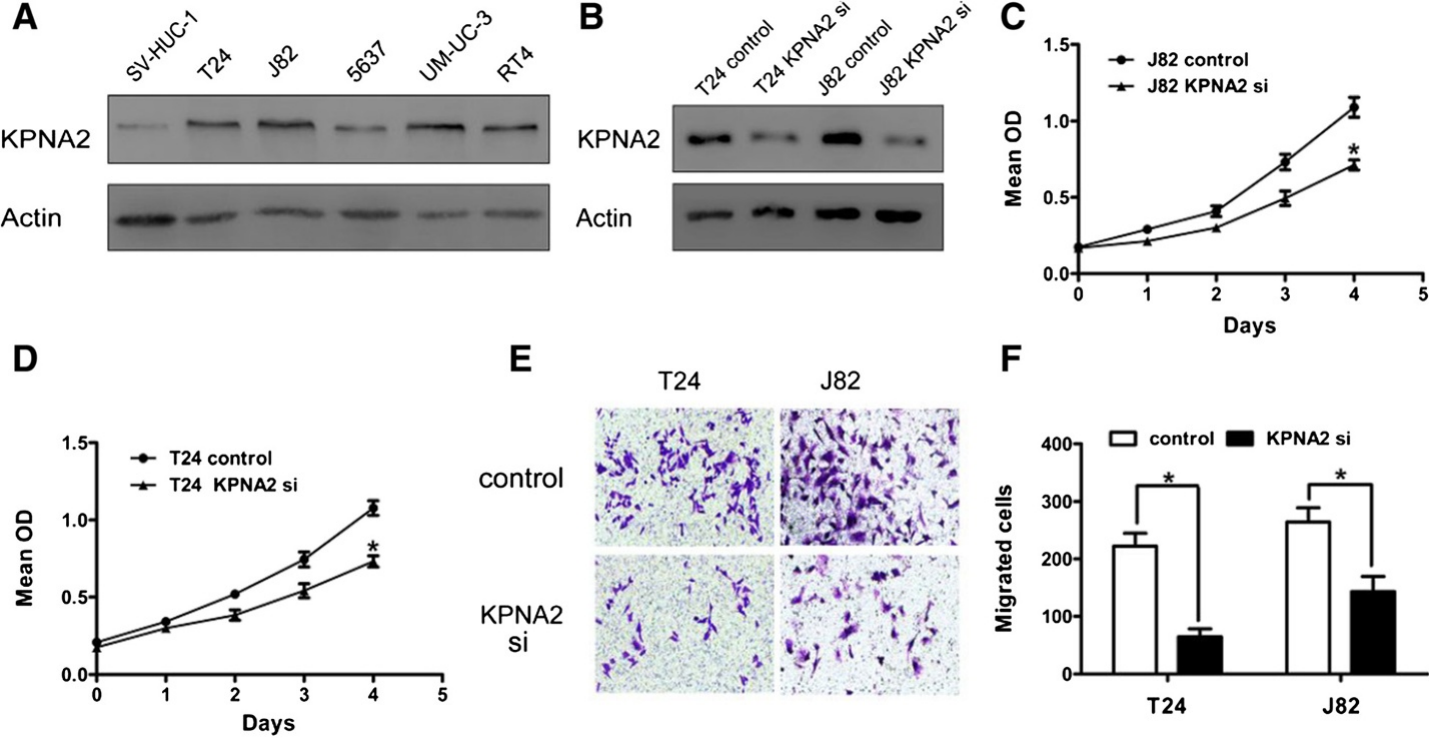
KPNA2 expression in urinary tract TCC cell lines and KPNA2 knockdown *in vitro*. **(A)** Western blot analyses of KPNA2 expression in normal and TCC cell lines of the urinary tract. **(B)** Western blot analyses of KPNA2 expression in T24 and J82 cells transfected with a control or KPNA2-directed siRNA. **(C, D)** Proliferation assay results from J82 and T24 cells transfected with a control or KPNA2-directed siRNA. **(E)** Representative images of migrating cells evaluated by the Boyden chamber assay (Original magnification: ×100). **(F)** The quantification results for migrating cells are presented as the mean ± SD of three independent experiments, with five random fields counted per chamber. *P < 0.05.

We employed *in vitro* techniques to investigate the mechanism by which KPNA2 contributes to UTUC malignancy. Consistent with the IHC staining results from UTUC specimens, western blot analysis showed that the KPNA2 level was very low in normal human urinary tract epithelial cell line SV-HUC-1, while cancer cell lines derived from low-grade (5637), superficial (RT4), and invasive (T24, J82, UM-UC-3, and EJ) urinary tract TCC showed increased levels of KPNA2 (Figure 2A). The T24 and J82 cell lines were selected for subsequent analysis because of their high KPNA2 expression levels among the urinary tract TCC cell lines.

siRNA technology was used to knockdown KPNA2 in T24 and J82 cells. Western blot analysis showed that following KPNA2-directed siRNA transfection, KPNA2 protein expression was significantly lower than in cells transfected with control RNA (Figure 2B). Thus, the KPNA2-directed siRNA was effective at silencing KPNA2 expression and was used for subsequent experiments.

Following knockdown of KPNA2, the viability of the cells was significantly lower than that of control cells, as assessed by a proliferation assay (Figure 2C and D). T24 and J82 cell migration was assayed by the Boyden chamber assay. As shown in Figure 2E and F, the number of migrating cells was significantly lower in KPNA2 knockdown cells than in control cells. These studies showed that knockdown of KPNA2 resulted in decreased proliferation and migration of urothelial carcinoma cells.

### KPNA2 knockdown induces apoptosis in urothelial carcinoma cells

**Figure 3:**
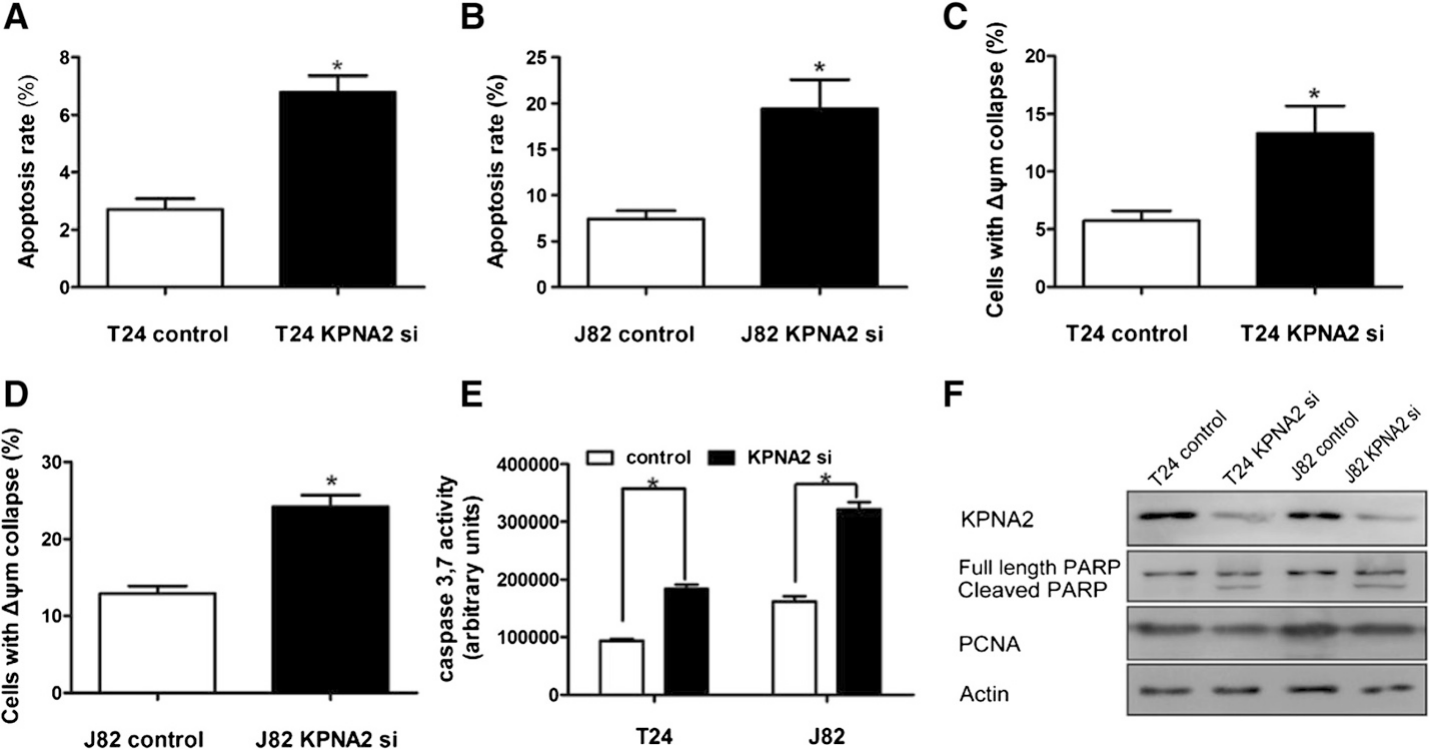
KPNA2 knockdown induces apoptosis in urothelial carcinoma cells. **(A**,**B)** The apoptosis rate, as evaluated by flow cytometry analysis using Annexin-V/PI staining. **(C**,**D)** The percentage of cells with mitochondrial Δψm disruption, as evaluated by flow cytometry analysis using the JC-1 dye. **(E)** Caspase 3/7 activity of T24 and J82 cells transfected with control or KPNA2-directed siRNA. The data in **(A**–**E)** represent the mean ± SD of three independent tests performed in triplicate. *P < 0.05. **(F)** Western blot analysis of PARP cleavage and PCNA expression in control and KPNA2 knockdown cells.

To assess whether increased apoptosis was involved in the significant decrease in cell viability following KPNA2 knockdown, we employed an apoptosis assay using Annexin V-FITC and propidium iodide double staining, followed by flow cytometry analysis. As shown in Figure 3A and B, the apoptosis rate in KPNA2 knockdown cells was significantly higher than in control cells. Consistently, KPNA2 knockdown induced a significant increase in the disruption of the mitochondrial Δψm compared with control cells, which was suggested to be an early event in the apoptotic process [22] [12766472] (Figure 3C, D).Furthermore, the activity of caspase-3 and −7, which are executioners of apoptosis, was also significantly increased after KPNA2 knockdown (Figure 3E). Consistently, the cleavage of PARP, a marker of apoptosis, was significantly increased in KPNA2 knockdown cells as demonstrated by a western blot assay (Figure 3F). The expression of PCNA, a marker of proliferation, was decreased in KPNA2 knockdown cells (Figure 3F). Taken together, these data show that KPNA2 knockdown activated the apoptosis pathway and decreased the proliferation of urothelial carcinoma cells.

## Discussion

To the best of our knowledge, this is the first study in which the relationships between expression of KPNA2 and the most clinically relevant features of UTUCs were evaluated. We demonstrated that KPNA2 was significantly upregulated in UTUC specimens. Moreover, high nuclear KPNA2 immunoreactivity was identified as a novel predictor of bladder recurrence and poor DFS and OS of UTUC patients after RNU, and its predictive ability was independent of the conventional predictive factors such as sex, tumor location, tumor size, and tumor multiplicity. Additionally, KPNA2 knockdown resulted in decreased cell proliferation and migration and increased apoptosis in urothelial carcinoma cells.

The karyopherins are an evolutionarily conserved family of transport factors that mediate the nucleocytoplasmic transport of large complexes (>40 kDa) [23]. The karyopherin family comprises both importins (import factors) and exportins (export factors). To date, approximately 22 importin β proteins and 6 importin α proteins have been identified in human cells [24]. Importin α/importin β heterodimers recognize cargo proteins based on their nuclear localization signal and mediate the classical nuclear protein import pathway [25]. KPNA2 (karyopherin α2) is a member of the karyopherin α family. It delivers numerous cargo proteins to the nucleus and is subsequently shuttled back to the cytoplasmic compartments through binding to Ran-GTP [26].

Identifying risk factors or biomarkers for bladder recurrence of UTUC would contribute greatly to the management of this disease due to its high incidence even after standard surgical procedures. However, there were no well-established prognostic factors until recently. Li and Chen et al. reported that male sex is an independent risk factor for bladder recurrence of Chinese a nd Taiwanese patients with UTUC after RNU [4,27]. Ureteric tumor location is also an independent predictor of bladder recurrence [28,29] and the development of muscle-invasive bladder cancer after RNU [30]. The tumor multiplicity of UTUC was shown to independently influence bladder recurrence after RNU in multiple studies [31]. In our study, KPNA2 immunoreactivity was demonstrated to be a prognostic factor for bladder recurrence of primary UTUC after RNU in the multivariate Cox regression analysis after excluding patients with bilateral synchronous UTUCs and those with previous or concomitant bladder tumors. KPNA2 immunoreactivity was independent of conventional factors, such as sex, tumor multiplicity, and tumor location. To date, none of the published biomarkers for UTUC has been utilized in clinical practice. The description of KPNA2 as an independent prognostic factor for bladder recurrence and survival clearly supports its expression as a promising prognosis biomarker for UTUC.

KPNA2 may affect oncogenesis by mediating the subcellular localization of cancer-associated cargo proteins. For example, KPNA2 controls the import of NBS1, a key component of the MRE11/RAD50/NBS1 complex that is involved in processing double-strand breaks, DNA recombination, and the maintenance of genomic stability [32]. NBS1 plays opposing roles in carcinogenesis based on its subcellular localization and is mainly regulated by KPNA2. Cytoplasmic NBS1 promotes tumorigenesis by activating the PI3-kinase/AKT pathway, while nuclear NBS1 acts as a tumor suppressive protein involved in DNA repair and cell cycle checkpoint control [33]. The NF-κB family member p65 (RelA) is also a cargo protein of KPNA2 [34]. Nuclear translocation of NF-κB p65 can promote carcinogenesis by increasing proliferation and migration and by activating the antiapoptotic pathway in cancer cells. Moreover, NF-κB p65 nuclear immunoreactivity is increased in UTUC tissues and is an independent predictor for disease-free survival and OS [35]. Therefore, the inhibited proliferation and migration and the increased apoptosis after KPNA2 silencing in our *in vitro* model may be due to the decreased nuclear translocation of NF-κB p65 by KPNA2. However, further studies are needed to fully illustrate the reciprocal effects between KPNA2 expression and p65-signaling in UTUC.

Our study has some limitations. First, it is limited by its single-institution and retrospective study design and the relatively short follow-up duration. Future prospective validation studies should be performed across multiple centers. Second, the present study does not include information on other risk factors, such as concomitant carcinoma in situ, urine cytology or surgical modality, which may improve the accuracy of bladder recurrence and survival predictions. Third, in the production of tissue microarrays, because of the limitations of pathologic specimens, we did not choose the tumor specimens whose diameter less than 1 cm, and that maybe cause selection bias.Additionally, our study focused on immunoreactivity of KPNA2 by IHC, thus, we did not fully evaluate the associations between KPNA2 immunoreactivity and other reported biomarkers, such as serum C-reactive protein levels [36], or KPNA2 cargo proteins, such as NF-κB p65 or NBS1.

## Conclusions

In conclusion, our results demonstrate that KPNA2 is overexpressed in UTUC specimens. Moreover, in this study, high expression of KPNA2 was a novel independent predictor for bladder recurrence and poor DFS and OS of UTUC patients after RNU.Further functional studies and prospective validation studies are needed to determine whether KPNA2 is a suitable therapeutic target or whether its high expression can be used as a novel risk factor when selecting UTUC patients who require more aggressive treatment regimens in the clinic.

### Ethical statement

This study was approved by the Ethic Commette of the Peking University First Hospital (Beijing, P.R. China).

## Abbreviations

KPNA2: Karyopherin alpha 2
UTUC: Upper tract urothelial carcinoma
TCC: Transitional cell carcinoma
RNU: Radical nephroureterectomy
siRNA: small interfering RNA
TMA: Tissue microarray
BMI: Body mass index
eGFR: estimated glomerular filtration rate
DFS: Disease-free survival
OS: Overall survival
JC-1: 5, 5′, 6, 6′-tetrachloro-1, 1′, 3, 3′-tetraethylbenzimidazolocarboc-yanine iodide
